# α‐Melanocyte‐stimulating hormone inhibition of oxytocin neurons switches to excitation in late pregnancy and lactation

**DOI:** 10.14814/phy2.15226

**Published:** 2022-03-21

**Authors:** Michael R. Perkinson, Matthew K. Kirchner, Meng Zhang, Rachael A. Augustine, Javier E. Stern, Colin H. Brown

**Affiliations:** ^1^ Brain Health Research Centre University of Otago Dunedin Aotearoa New Zealand; ^2^ Centre for Neuroendocrinology University of Otago Dunedin Aotearoa New Zealand; ^3^ Department of Physiology University of Otago Dunedin Aotearoa New Zealand; ^4^ Center for Neuroinflammation and Cardiometabolic Diseases Georgia State University Atlanta Georgia USA

**Keywords:** lactation, oxytocin, pregnancy, somato‐dendritic secretion, vasopressin

## Abstract

Oxytocin is secreted into the periphery by magnocellular neurons of the hypothalamic supraoptic and paraventricular nuclei (SON and PVN) to trigger uterine contraction during birth and milk ejection during suckling. Peripheral oxytocin secretion is triggered by action potential firing, which is regulated by afferent input activity and by feedback from oxytocin secreted into the extracellular space from magnocellular neuron somata and dendrites. A prominent input to oxytocin neurons arises from proopiomelanocortin neurons of the hypothalamic arcuate nucleus that secrete an alpha‐melanocyte‐stimulating hormone (α‐MSH), which inhibits oxytocin neuron firing in non‐pregnant rats by increasing somato‐dendritic oxytocin secretion. However, α‐MSH inhibition of oxytocin neuron firing is attenuated in mid‐pregnancy and somato‐dendritic oxytocin becomes auto‐excitatory in late‐pregnancy and lactation. Therefore, we hypothesized that attenuated α‐MSH inhibition of oxytocin neuron firing marks the beginning of a transition from inhibition to excitation to facilitate peripheral oxytocin secretion for parturition and lactation. Intra‐SON microdialysis administration of α‐MSH inhibited oxytocin neuron firing rate by 33 ± 9% in non‐pregnant rats but increased oxytocin neuron firing rate by 37 ± 12% in late‐pregnant rats and by 28 ± 10% in lactating rats. α‐MSH‐induced somato‐dendritic oxytocin secretion measured *ex vivo* with oxytocin receptor‐expressing “sniffer” cells, was of similar amplitude in PVN slices from non‐pregnant and lactating rats but longer‐lasting in slices from lactating rats. Hence, α‐MSH inhibition of oxytocin neuron activity switches to excitation over pregnancy while somato‐dendritic oxytocin secretion is maintained, which might enhance oxytocin neuron excitability to facilitate the increased peripheral secretion that is required for normal parturition and milk ejection.

## Graphical abstract

Alpha‐MSH activation of somato‐dendritic oxytocin secretion is maintained in pregnancy and lactation but the released oxytocin switches from auto‐inhibition to auto‐excitation to support birth and lactation.

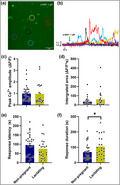

## 1 INTRODUCTION

In mammals, delivery and feeding of the newborn requires the nonapeptide, oxytocin, which is synthesized and secreted by magnocellular neurons of the supraoptic nucleus (SON) and paraventricular nucleus (PVN) in the hypothalamus. Oxytocin secretion into the circulation is triggered by action potential (spike) invasion of the axon terminals of the posterior pituitary gland. Over the course of pregnancy, the oxytocin system is modified to prepare for increased secretion necessary for uterine contraction during parturition and milk ejection during suckling. While intrinsic changes occur in oxytocin neurons over pregnancy to facilitate increased activity (Armstrong et al., 2002; Perkinson et al., 2021), changes in neuronal and hormonal inputs also contribute to increased oxytocin secretion at parturition and in lactation (Augustine et al., 2017; Brussaard et al., 1997; Israel & Poulain, 2000; Oliet et al., 2001; Stern et al., 2000).

In addition to axon terminal secretion, oxytocin neurons also secrete oxytocin from their somata and dendrites (somato‐dendritic secretion) to modulate their own excitability. Oxytocin neurons express oxytocin receptor mRNA (Meddle et al., 2007) and oxytocin receptor activation increases intracellular calcium (Lambert et al., 1994) to decrease the firing rate of oxytocin neurons by triggering endocannabinoid release for retrograde inhibition of glutamatergic synaptic transmission (Hirasawa et al., 2004; Kombian et al., 1997). However, the activation of oxytocin receptors switches to excitation of oxytocin neurons in lactation (Freund‐Mercier & Richard, 1984; Moos et al., 1989a). Furthermore, oxytocin receptor mRNA expression increases in oxytocin neurons over pregnancy (Meddle et al., 2007) and somato‐dendritic oxytocin release increases immediately before each burst of action potential firing in oxytocin neurons during lactation (Moos et al., 1989a). Hence, it appears that regulation of oxytocin neuron activity by somato‐dendritic oxytocin secretion changes over pregnancy to facilitate increased axon terminal secretion of oxytocin into the circulation for birth and lactation.

While both depend on intracellular calcium, axon terminal secretion and somato‐dendritic secretion can occur independently from oxytocin neurons, suggesting that different mechanisms control secretion from different compartments of the neurons (Pitra et al., 2019), which would allow for differential modulation of the two modes of secretion by afferent inputs (Ludwig & Leng, 2006). Alpha‐melanocyte‐stimulating hormone (α‐MSH) is synthesized by proopiomelanocortin (POMC) neurons of the hypothalamic arcuate nucleus and has been shown to differentially modulate axon terminal secretion and somato‐dendritic secretion of oxytocin. Oxytocin neurons express receptors for α‐MSH (MC4R) and receive inputs from POMC neurons (Douglas et al., 2002). In non‐pregnant rats, MC4R activation inhibits peripheral oxytocin secretion through reduced action potential firing and simultaneously increases somato‐dendritic oxytocin secretion via increased intracellular calcium (Sabatier et al., 2003). However, α‐MSH inhibition of oxytocin neuron activity is lost by mid‐pregnancy (Ladyman et al., 2016) and it is unknown whether α‐MSH stimulation of somato‐dendritic oxytocin is modulated by reproductive status.

Therefore, we hypothesized that attenuation of α‐MSH inhibition of oxytocin neuron activity in mid‐pregnancy represents the beginning of a transition from inhibition to excitation to facilitate peripheral oxytocin secretion for parturition and lactation and that α‐MSH stimulation of somato‐dendritic oxytocin secretion would be maintained to support enhanced peripheral oxytocin secretion for parturition and lactation. Consistent with our hypotheses, we found that intra‐SON administration of α‐MSH inhibited oxytocin neurons in non‐pregnant rats but excited oxytocin neurons in late‐pregnant and lactating rats and that α‐MSH‐induced somato‐dendritic oxytocin secretion induced similar responses in oxytocin receptor‐expressing “sniffer” (sniffer_OT_) cells in PVN slices from non‐pregnant and lactating rats.

## 2 METHODS

### 2.1 Ethics approvals

Electrophysiology procedures were approved by the University of Otago Animal Ethics Committee and were carried out in accordance with the New Zealand Animal Welfare Act and associated guidelines. Sniffer cell procedures were approved by Georgia State University Institutional Animal Care and Use Committee and were carried out in accordance with NIH guidelines.

### 2.2 Animals

For electrophysiology, 10‐week‐old, female Sprague–Dawley rats were purchased from the University of Otago Animal Facility. Rats were group‐housed until mid‐pregnancy (gestation day 14, G14) then housed individually until the day of the experiment. Rats were kept in controlled light conditions (12 h–12 h; lights on at 07.00 h; 22 ± 1°C) with ad libitum access to standard rat chow and tap water. To generate late‐pregnant and lactating rats, daily vaginal smears were taken to monitor the estrous cycle (Hubscher et al., 2005). Following identification of a pro‐estrous smear, rats were housed overnight with a male rat and sperm present in the next morning's smear was determined as G0. Lactating rats typically gave birth on days 21–22 of gestation (post‐partum day 1; PP1).

For sniffer cells experiments, non‐pregnant Sprague–Dawley rats were group‐housed under controlled conditions (light‐dark cycle [12 h–12 h; 22–24°C]), with ad libitum access to standard rat chow and tap water. Lactating rats were purchased from the Charles River Laboratory and delivered for use on PP14.

In all experiments, non‐pregnant rats were freely‐cycling to avoid any confounding effect of the estrous cycle.

### 2.3 In vivo electrophysiology

On the day of electrophysiology, non‐pregnant, late‐pregnant (G18–G21) or lactating (PP7–PP17) rats were anesthetized with 1.25 g kg^−1^ I.P. urethane (ethyl carbamate, Sigma, St Louis, MO, USA). Once cessation of the pedal withdrawal reflex was confirmed, the left femoral vein was catheterized to allow systemic administration of (Tyr[SO_3_H]^27^)cholecystokinin fragment 26–33 amide (CCK8S; Sigma). The pituitary stalk and right SON were exposed by transpharyngeal surgery (Brown et al., 2014). A U‐shaped microdialysis probe (permeable to 10 kDa; in‐house design (Horn & Engelmann, 2001)) was placed onto the ventral surface of the brain.

Extracellular single‐unit recordings were made with a glass recording microelectrode (15–40 MΩ; filled with 0.9% saline) connected to a Neurolog system (Digitimer Ltd, UK). A side‐by‐side SNEX‐200 bipolar stimulating electrode (Science Products GmbH, Hofheim, Germany) was placed on the pituitary stalk to depolarize the axons and elicit antidromic action potentials in SON neurons. Neuronal activity was recorded via a CED 1401 analog–digital interface (Cambridge Electronic Design) using Spike 2 software (Cambridge Electronic Design) and analyzed offline. Neurons were characterized as oxytocin neurons on the basis of a transient excitation of greater than 0.5 spikes s^−1^ averaged over 5 min following IV CCK8S injection (20 µg kg^−1^, 0.5 ml kg^−1^ in 0.9% saline) (Brown et al., 1996), or as vasopressin neurons by transient inhibition, or no effect, following CCK8S injection (Scott et al., 2009). The SON was continuously dialyzed with artificial cerebrospinal fluid (aCSF; mmol L^−1^: NaCl 138, KCl 3.36, NaHCO_3_ 9.52, Na_2_HPO_4_ 0.49, urea 2.16, CaCl_2_ 1.26, MgCl_2_ 1.18; osmolality 295–300 mosmol kg^−1^) and switched to α‐MSH in aCSF (Sigma; 1.5 mM) at 3 μl min^−1^ for 30 min. At the end of the experiment, the rats were euthanized by IV injection of 0.5 ml KCl (3 mol L^−1^).

### 2.4 Slice preparation for sniffer_OT_ cells

Hypothalamic brain slices were prepared as previously described (Pitra et al., 2019; Son et al., 2013). Briefly, rats were anesthetized with pentobarbital (50 mg kg^−1^ I.P.). Once fully anesthetized, rats were transcardially perfused with ice‐cold sucrose aCSF containing (in mM): 2.5 KCl, 1 MgSO_4_, 26 NaHCO_3_, 1.25 NaH_2_PO_4_, 20 D‐glucose, 0.4 ascorbic acid, 2 CaCl_2_, and 210 sucrose; pH 7.3; 295 mosm. The brain was dissected out and coronal slices (240 μm) of the hypothalamus containing the PVN were cut in the same ice‐cold sucrose aCSF constantly bubbled with 95%O_2_/5%CO_2_. Once the brain slices were cut, they were transferred to a holding chamber containing standard aCSF warmed at 32°C for 20 min and then resting at room temperature for at least 40 min before the start of the experiment.

### 2.5 Sniffer_OT_ cells

Full methods are as described in Pitra et al. (2019). Briefly, sniffer_OT_ cells were generated by culturing Chinese hamster ovary (CHO) cells in Dulbecco's Modified Eagle Medium containing 10% w/v fetal bovine serum, 1% w/v penicillin–streptomycin, 1% w/v Na‐Pyruvate, and 1% w/v NaCO_3_ filtered once through a Nalgene filtration system, and transfecting with pcDNA3.1+ containing human oxytocin receptors cloned in EcoRI (5’) and XhoI (3’) (plasmid obtained from Missouri S&T cDNA Resource Center, Rolla, MO, USA) using lipofectamine, and stable overexpression was achieved by geneticin (500 mg ml^−1^) selection (Piñol et al., 2014). Sniffer_OT_ cells were then plated and transiently transfected to express the red fluorescent genetically encoded calcium indicator (R‐GECO; GenScript, Piscataway, NJ, USA) with Fugene HD reagent (Promega, Madison, WI, USA).

Sniffer_OT_ cells were resuspended in standard aCSF ([in mmol L^−1^]: 119 NaCl, 2.5 KCl, 1 MgSO_4_, 26 NaHCO_3_, 1.25 NaH_2_PO_4_, 20 D‐glucose, 0.4 ascorbic acid, 2 CaCl_2_, and 2 pyruvic acid; pH 7.3; 295 mosm) with trypsin (0.05%). Sniffer_OT_ cells were transferred directly onto the lateral magnocellular subdivision of the PVN in brain slices after pausing aCSF superfusion. After at least 5 min, aCSF superfusion was resumed for 5 min to wash off any unattached sniffer_OT_ from the slice before proceeding with the recording. Sniffer_OT_ cells adopted a rounded appearance when transferred to aCSF and no further overt morphological changes were observed during the equilibration period on the brain slices. Experiments were restricted to preparations that had at least five fluorescently visible sniffer cells in the field (~10 on average).

### 2.6 Imaging and analysis of the calcium changes in sniffer_OT_ cells

To record the calcium‐induced fluorescence changes of sniffer_OT_ cells, images were taken with the Andor Technology Revolution system (iXON EMCCD camera with the Yokogawa CSU 10 [Tokyo, Japan], confocal scanning unit; Belfast, UK), at a rate of 4 Hz. The sniffer_OT_ cell fluorescence was imaged under a 488 nm excitation light and the calcium response was measured at >495 nm (Fluo‐5F). To test for somato‐dendritic secretion of oxytocin in response to α‐MSH, hypothalamic slices were constantly superfused with aCSF at 32°C. Slices were stimulated with a 1 ml bolus of α‐MSH (1 µM; concentration on slice ~0.1 µM) and then with 1 ml oxytocin (10 µM; concentration on slice ~1 µM; at least 10 min after α‐MSH). Drugs were pumped into the aCSF line by hand at ~0.1 ml s^−1^ and fluorescent calcium responses were monitored in surrounding sniffer_OT_ cells. Responses to α‐MSH are reported only from snifferOT cells that responded to exogenous oxytocin with an increase in fluorescence of >20% from baseline.

Each slice was imaged separately. Imaging data were analyzed using ImageJ software (NIH). For quantitative measurements, fractional fluorescence (F/F_0_) was determined by dividing the fluorescence intensity (F) within a region of interest by a baseline fluorescence value (F_0_) determined from 30 frames/images before stimulation (Stern & Potapenko, 2013). Peak calcium amplitude was the maximum F/F_0_ achieved after α‐MSH. Latency to the response was determined as the time between the start of the α‐MSH bolus and the start of the calcium response of each sniffer cell. Response duration was the duration from the start of the response to the return baseline. The area under the curve was calculated for the duration of the response. Response rates are the number of cells that responded to both a‐MSH and oxytocin relative to all cells that responded to oxytocin. Sniffer_OT_ cells that showed intrinsic oscillatory calcium activity were excluded from the analysis. To better display changes in fluorescence levels, images were pseudocolored using ImageJ.

### 2.7 Data analysis and statistics

Hazard function analysis was done on the 10 min pre‐α‐MSH and last 10 min of α‐MSH administration (Methods and Results in Supplementary Figures S1 and S2). All values are reported as mean ± standard error of the mean (SEM). Statistical analyses were completed using Sigma Plot version 12 for Windows (Systat Software, San Jose, CA, USA) or Prism version 9.1.0 (GraphPad Software, San Diego, CA, USA). One‐way or two‐way ANOVA was used to compare multiple groups; where the F‐ratio was significant, ANOVA was followed by all‐pairwise Holm–Sidak post hoc tests. The Student's *t*‐test was used to compare sniffer cell data. *p* < 0.05 was considered significant.

## 3 RESULTS

### 3.1 Spontaneous firing rate of magnocellular neurons was similar in all reproductive states

Spontaneous firing rate was analyzed from the 10 min prior to α‐MSH for 33 oxytocin neurons and 24 vasopressin neurons from 13 non‐pregnant rats, ten late‐pregnant rats and 17 lactating rats. There was no difference between the firing rate of oxytocin neurons (F_2,29_ = 0.18, *p* = 0.84, one‐way ANOVA) in non‐pregnant (5.23 ± 0.72 spikes s^−1^, *n* = 10), late‐pregnant (5.19 ± 1.40 spikes s^−1^, *n* = 7) and lactating rats (4.59 ± 0.70 spikes s^−1^, *n* = 15). Vasopressin neuron firing rate was also similar (F_2,21_ = 1.37, *p* = 0.28) between non‐pregnant (6.11 ± 0.60 spikes s^−1^, *n* = 10), late‐pregnant (6.92 ± 0.80 spikes s^−1^, *n* = 6), and lactating rats (5.08 ± 0.85 spikes s^−1^, *n* = 8).

### 3.2 Alpha‐melanocyte‐stimulating hormone inhibits oxytocin neurons in non‐pregnant rats but excites oxytocin neurons in late‐pregnant and lactating rats

**FIGURE 1:**
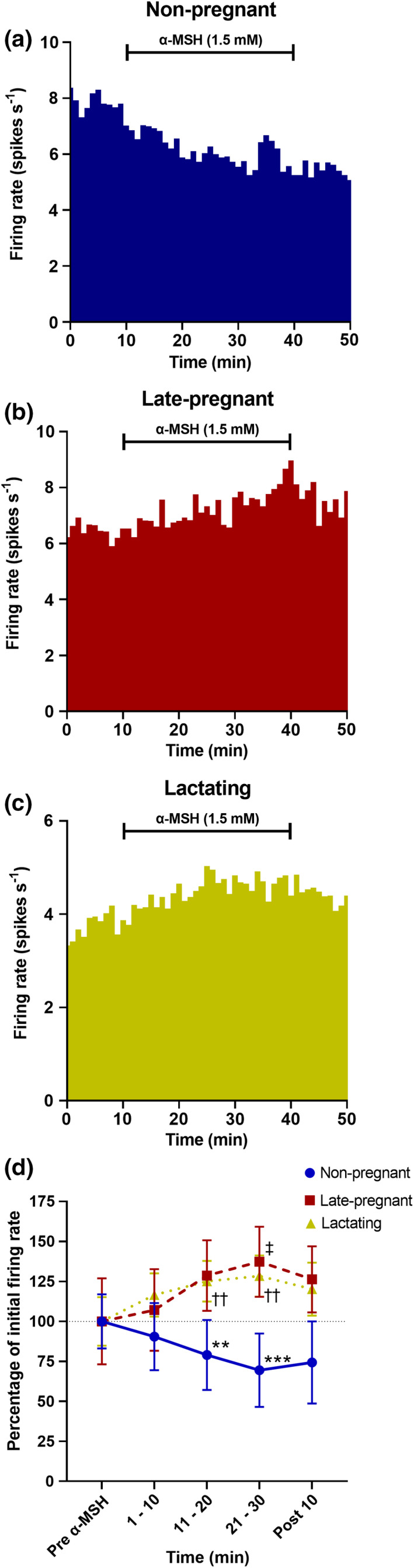
The effect of α‐MSH on the firing rate of oxytocin neurons is dependent on reproductive status. (a–c) Example ratemeter recordings (in 1 min bins) of oxytocin neurons during microdialysis alpha‐melanocyte‐stimulating hormone (α‐MSH; 1.5 mM) administration for 30 min in non‐pregnant (a), late‐pregnant (b) and lactating (c) urethane‐anesthetized rats. Microdialysis α‐MSH administration was maintained for 30 min in 11 oxytocin neurons from ten non‐pregnant rats, seven oxytocin neurons from seven late‐pregnant rats (G18–G21) and 15 oxytocin neurons from 13 lactating rats (PP7–L17). (d) Percentage change in firing rate ± SEM (in 10 min bins) of oxytocin neurons during 30 min microdialysis α‐MSH administration compared to the initial firing rate prior to α‐MSH administration. Administration of α‐MSH caused a change in firing rate of oxytocin neurons that was dependent on the reproductive status of the rats (REPRODUCTIVE STATUS [RS]: F_2,29_ = 0.34, *p* = 0.71; TIME: F_2,29_ = 0.43, *p* = 0.66; interaction between RS and TIME: F_6,87_ = 11.249, *p* < 0.001, two‐way repeated measures ANOVA). **p = 0.002 and ***p < 0.001 compared to pre‐α‐MSH in non‐pregnant rats, ‡*p* = 0.014 compared to pre‐α‐MSH in late‐pregnant rats, ††*p* = 0.002 compared to pre‐α‐MSH in lactating rats, Holm–Sidak post hoc tests. However, there were no differences in the firing rate of oxytocin neurons between non‐pregnant, late‐pregnant and lactating rats at any time point

To determine whether α‐MSH induced changes in oxytocin neuron firing rate is affected by reproductive status, *in vivo* electrophysiological recordings were maintained during α‐MSH administration for 30 min in ten oxytocin neurons from nine non‐pregnant rats, seven oxytocin neurons from seven late‐pregnant rats, and 15 oxytocin neurons from 14 lactating rats. The effect of intra‐SON α‐MSH administration on oxytocin neuron firing rate was dependent on reproductive status (interaction between REPRODUCTIVE STATUS and TIME F_(6, 87)_ = 11.25, *p* < 0.001, two‐way RM ANOVA; Figure 1). Consistent with previous observations, α‐MSH administration progressively decreased oxytocin neuron firing rate in non‐pregnant rats (*p* < 0.001, Holm–Sidak test). In contrast to non‐pregnant rats, α‐MSH administration progressively increased the firing rate of oxytocin neurons in late‐pregnant rats (*p* = 0.014) and lactating rats (*p* = 0.002).

### 3.3 Alpha‐melanocyte‐stimulating hormone has no effect on the firing rate of vasopressin neurons in non‐pregnant, late‐pregnant, or lactating rats

**FIGURE 2:**
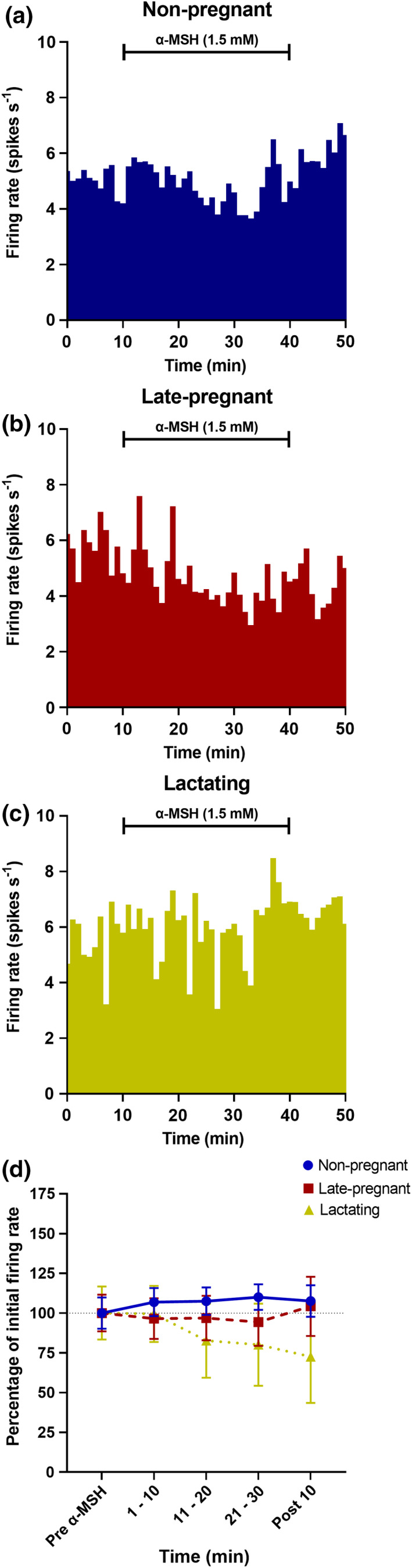
α‐MSH has no effect on the firing rate of vasopressin neurons. (a–c) Example ratemeter recordings (in 1 min bins) of vasopressin neurons during microdialysis alpha‐melanocyte‐stimulating hormone (α‐MSH; 1.5 mM) administration for 30 min in non‐pregnant (a), late‐pregnant (b) and lactating (c) urethane‐anesthetized rats. Microdialysis α‐MSH administration was maintained for 30 min in 10 vasopressin neurons from seven non‐pregnant rats, six vasopressin neurons from five late‐pregnant rats (G18–G20) and eight vasopressin neurons from seven lactating rats (PP7–L16). (d) Percentage change in firing rate ± SEM (in 10 min bins) of vasopressin neurons during 30 min microdialysis α‐MSH administration compared to the initial firing rate prior to α‐MSH administration. Administration of α‐MSH had no effect on the firing rate of vasopressin neurons recorded from non‐pregnant, late‐pregnant or lactating rats (RS: F_2,21_ = 0.16, *p* = 0.23; TIME: F_2,21_ = 0.65, *p* = 0.57; interaction between RS and TIME: F_6,63_ = 1.24, *p* = 0.30, two‐way repeated measures ANOVA)

To test whether the α‐MSH effects on firing rate were specific to oxytocin neurons, we made *in vivo* electrophysiological recordings during α‐MSH administration in ten vasopressin neurons from seven non‐pregnant rats, six vasopressin neurons from five late‐pregnant rats, and eight vasopressin neurons from seven lactating rats. Vasopressin neuron firing rate was unaffected by α‐MSH administration (interaction between REPRODUCTIVE STATUS and TIME: F_6, 63_ = 1.24, *p* = 0.30, two‐way RM ANOVA, Figure 2).

### 3.4 Alpha‐melanocyte‐stimulating hormone‐induced similar sniffer_OT_ cell calcium responses in PVN slices from non‐pregnant and lactating rats

**FIGURE 3:**
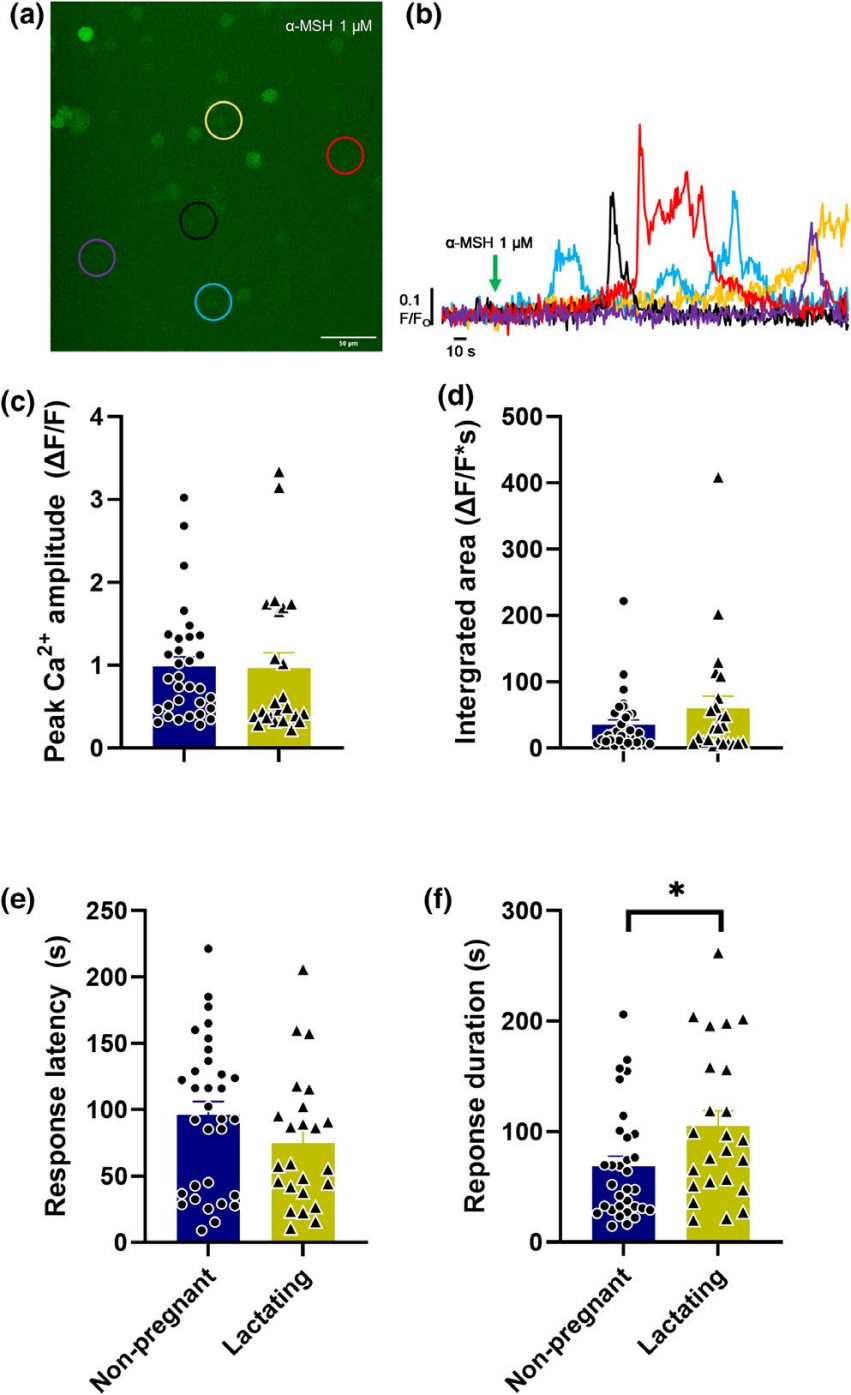
Somato‐dendritic oxytocin secretion in response to α‐MSH administration is similar in hypothalamic slices from non‐pregnant and lactating rats. (a). Example image of calcium responses (F/F_0_) from sniffer_OT_ cells plated onto a hypothalamic slice containing the paraventricular nucleus from a non‐pregnant rat. The circles indicate cells that had fluorescence measured shown in (b). (b) The coloured lines reflect the change in calcium response (F/F_0_) of the five individual cells from one recording (these changes can be seen in the video; Figure S3). The arrow indicates the start of the α‐MSH (1 µM) bolus. (c–f) Summary data of the calcium responses from sniffer_OT_ cells displaying the peak amplitude (c), integrated area under the Ca^2+^ response curve (d), latency to start of response (e) and duration of the calcium response (f) (*p = 0.027, unpaired Student's *t*‐test). Data contains 32 oxytocin responsive sniffer_OT_ cells plated onto hypothalamic slices from three non‐pregnant rats and 24 oxytocin responsive sniffer_OT_ cells plated onto hypothalamic slices from two lactating rats

To test whether α‐MSH stimulated somato‐dendritic oxytocin secretion is maintained during lactation, sniffer_OT_ cells were plated onto hypothalamic slices containing the PVN and the calcium response induced by exogenous α‐MSH was recorded. α‐MSH was superfused onto slices from three non‐pregnant rats (12 slices) and two lactating rats (12 slices). The proportion of sniffer_OT_ cells that responded to α‐MSH was similar in slices from non‐pregnant rats (61.5%; 32/52) and lactating rats (72.7%; 24/33; Chi‐square = 0.45, *p* = 0.50). There was no difference in the peak amplitude (*p* = 0.93, Student's *t*‐test), the area under the curve (*p* = 0.17) or latency to response after α‐MSH between slices from non‐pregnant rats and lactating rats (*p* = 0.16; Figure 3). However, there was a longer α‐MSH‐induced response duration in sniffer_OT_ cells on slices from lactating rats than in non‐pregnant rats (*p* = 0.03).

## 4 DISCUSSION

Here, we found that α‐MSH inhibition of oxytocin neurons switches to excitation in late‐pregnant rats and that this switch to excitation is maintained into lactation. We also found that α‐MSH‐induced somato‐dendritic oxytocin secretion is maintained in lactating rats, with a modest increase in the duration of the calcium response in sniffer_OT_ cells on slices from lactating rats. While the prolonged sniffer_OT_ cell response could result from subtle differences in washout between brain slice preparations and/or reduced breakdown of oxytocin in the extracellular space in lactation, the tortuosity of the extracellular space is decreased in the SON during lactation (Piet et al., 2004) and hypothalamic oxytocinase activity is consistently higher in lactating rats than in non‐pregnant rats (Tobin et al., 2014). Hence, the prolonged sniffer_OT_ cell response likely results from increased α‐MSH‐induced somato‐dendritic oxytocin secretion in lactation, rather than reduced oxytocin breakdown.

While the somato‐dendritic oxytocin secretion triggered by exogenous α‐MSH was only modestly higher in slices from lactating rats than in slices from non‐pregnant rats, it is possible that the stimulation by endogenous α‐MSH in lactation is more prominent than the endogenous inhibition before pregnancy because arcuate nucleus POMC mRNA expression increases in over the course of pregnancy (Douglas et al., 2002). Indeed, oxytocin levels rise in the SON during suckling in rats (Moos et al., 1989b). Hence, plasticity in α‐MSH modulation of oxytocin neurons appears to be part of the suite of physiological adaptations required to prepare the system for the increased oxytocin secretion necessary for successful parturition and lactation.

α‐MSH inhibits oxytocin neuron firing rate in non‐pregnant rats by increasing somato‐dendritic oxytocin secretion to trigger retrograde inhibition of synaptic transmission via endocannabinoid activation of cannabinoid 1 (CB1) receptors on excitatory axon terminals (Sabatier & Leng, 2006; Sabatier et al., 2013). The mechanism that underpins the switch to α‐MSH stimulation of oxytocin neuron firing rate in late‐pregnant and lactating rats has yet to be determined but cannot be simply explained by receptor downregulation or desensitization. We used the hazard function to investigate changes in post‐spike excitability (Brown et al., 2007) of oxytocin neurons over reproduction during α‐MSH adminstration. While we found a change in the mean late hazard, which infers the influence of baseline membrane potential and ongoing synaptic input on spiking activity, it was not specific to any group (Figure S1). Hence, there appears to be no precise change in post‐spike excitability that accounts for the change in response of oxytocin neurons to α‐MSH over reproductive states. Rather, it presumably involves changes in the coupling of α‐MSH, oxytocin, and/or endocannabinoid signalling in the oxytocin system.

### 4.1 α‐MSH stimulation of somato‐dendritic oxytocin secretion

MC4R couples to multiple signaling pathways, including mobilization of intracellular calcium (Kumar et al., 2021), which presumably mediates α‐MSH stimulation of somato‐dendritic oxytocin secretion. Given that the sniffer_OT_ cell response to α‐MSH‐induced somato‐dendritic secretion was similar in slices from non‐pregnant and lactating rats, it appears likely that the switch from α‐MSH inhibition to excitation of oxytocin neurons is mediated by changes downstream of somato‐dendritic oxytocin secretion.

Oxytocin inhibits oxytocin neurons in brain slices from non‐pregnant rats (Kuriyama et al., 1993) but is auto‐excitatory during lactation, inducing depolarization (Kawarabayashi et al., 1993; Wang et al., 2006) and attenuating GABA inhibition (Brussaard et al., 1996), and intracerebroventricular oxytocin receptor antagonist administration inhibits peripheral oxytocin secretion during suckling (Lambert et al., 1993; Richard et al., 1991). Hence, direct oxytocin neuron excitation by α‐MSH‐induced somato‐dendritic oxytocin secretion might over‐ride ongoing retrograde inhibition of excitatory synaptic transmission by somato‐dendritic oxytocin‐induced endocannabinoids in lactation.

### 4.2 Retrograde endocannabinoid signaling from oxytocin neurons

While α‐MSH‐induced somato‐dendritic oxytocin secretion might directly excite oxytocin neurons in late pregnancy and lactation, it is possible that endocannabinoid signaling also switches from inhibitory to excitatory. In addition to CB1 receptors, endocannabinoids can also activate transient receptor potential vanilloid (TRPV) receptors (Branco & Staras, 2009) that allow non‐specific cation influx to induce depolarization (Nilius et al., 2007). ΔN‐truncated TRPV1 (ΔN‐TRVP1) mRNA is expressed in oxytocin neurons but these do not contribute to the basal activity of oxytocin neurons in non‐pregnant and late‐pregnant rats (Perkinson et al., 2021). However, in the presence of a CB1 antagonist, α‐MSH causes a small excitation of oxytocin neurons in non‐pregnant rats (Sabatier & Leng, 2006). While CB1 receptors are still functional during lactation (Vilela & Giusti‐Paiva, 2014), if CB1 receptor expression on excitatory inputs to oxytocin neurons decreases in lactation, the balance of endocannabinoid effects might shift from inhibition to excitation during lactation.

### 4.3 Somato‐dendritic oxytocin release and food intake in pregnancy and lactation

In addition to feedback regulation of oxytocin neuron activity, somato‐dendritic oxytocin secretion has been implicated in mediating α‐MSH’s satiety effects via activation of ventromedial hypothalamic neurons (Sabatier et al., 2013). During pregnancy and lactation, changes in the central control of satiety cause hyperphagia to cope with the metabolic demands of pregnancy and lactation (Trujillo et al., 2011). ICV oxytocin inhibits food intake in non‐pregnant rats but increases food intake in mid‐pregnant rats (Douglas et al., 2007). Hence, the maintenance of α‐MSH‐induced somato‐dendritic oxytocin secretion might also help coordinate increased food intake while facilitating oxytocin secretion for parturition and lactation.

### 4.4 Alpha‐melanocyte‐stimulating hormone does not affect vasopressin neuron activity

Vasopressin is secreted by a distinct population of magnocellular neurons of the SON and PVN to trigger water reabsorption by the kidney in response to increased plasma osmolality. Vasopressin neurons also undergo adaptations over the course of pregnancy to increase blood volume to cope with the cardiovascular demands of pregnancy and lactation (Koehler et al., 1993; Prager‐Khoutorsky & Bourque, 2010). In non‐pregnant rats, vasopressin neurons do not express MC4R (Mountjoy et al., 1994) or respond to α‐MSH (Sabatier et al., 2003). We found that vasopressin neurons remain unresponsive to α‐MSH in late pregnancy and lactation, further supported by no change in the post‐spike excitability (Figure S2), suggesting that α‐MSH is not involved in the reproductive plasticity of vasopressin neurons.

## 5 CONCLUSION

Oxytocin neurons show dramatic plasticity in morphology, intrinsic properties, and responses to afferent inputs over the course of pregnancy to prepare for increased oxytocin secretion necessary for parturition and lactation. The present study adds a switch in α‐MSH inhibition to excitation to the suite of changes that promote oxytocin secretion for parturition and lactation. While α‐MSH‐induced somato‐dendritic oxytocin secretion was maintained in lactation to contribute to the excitation, the mechanisms by which is does so remain to be determined.

## TRANSLATIONAL PERSPECTIVE

Preterm birth is a major cause of infant mortality and lifelong morbidity. Appropriate activation of oxytocin neurons is critical for normal birth and early activation of oxytocin neurons can lead to preterm delivery. Our research shows that α‐MSH inhibition of oxytocin neurons switches to excitation in late pregnancy. Hence, antagonising α‐MSH activation of oxytocin neurons might provide a novel therapeutic target to reduce the risk of the preterm birth in at‐risk pregnancies.

## CONFLICT OF INTEREST

The authors have no competing financial interests.

## AUTHOR CONTRIBUTION

All authors contributed to the design and interpretation of the experiments. Michael R. Perkinson, Matthew K. Kirchner, Rachael A. Augustine, and Colin H. Brown performed the experiments. Michael R. Perkinson and Matthew K. Kirchner analyzed data. Michael R. Perkinson prepared the figures and the first draft of the manuscript. All authors reviewed the manuscript for intellectual content. The final version of manuscript for publication was explicitly approved by all authors.

## Supporting information



Fig S1

Fig S2

Video S1
