# Supplementary figures and images for: α‐Melanocyte‐stimulating hormone inhibition of oxytocin neurons switches to excitation in late pregnancy and lactation
Source: Physiol Rep. 2022 Mar 21;10(6):e15226. doi: 10.14814/phy2.15226 (PMC8935534; doi:10.14814/phy2.15226)

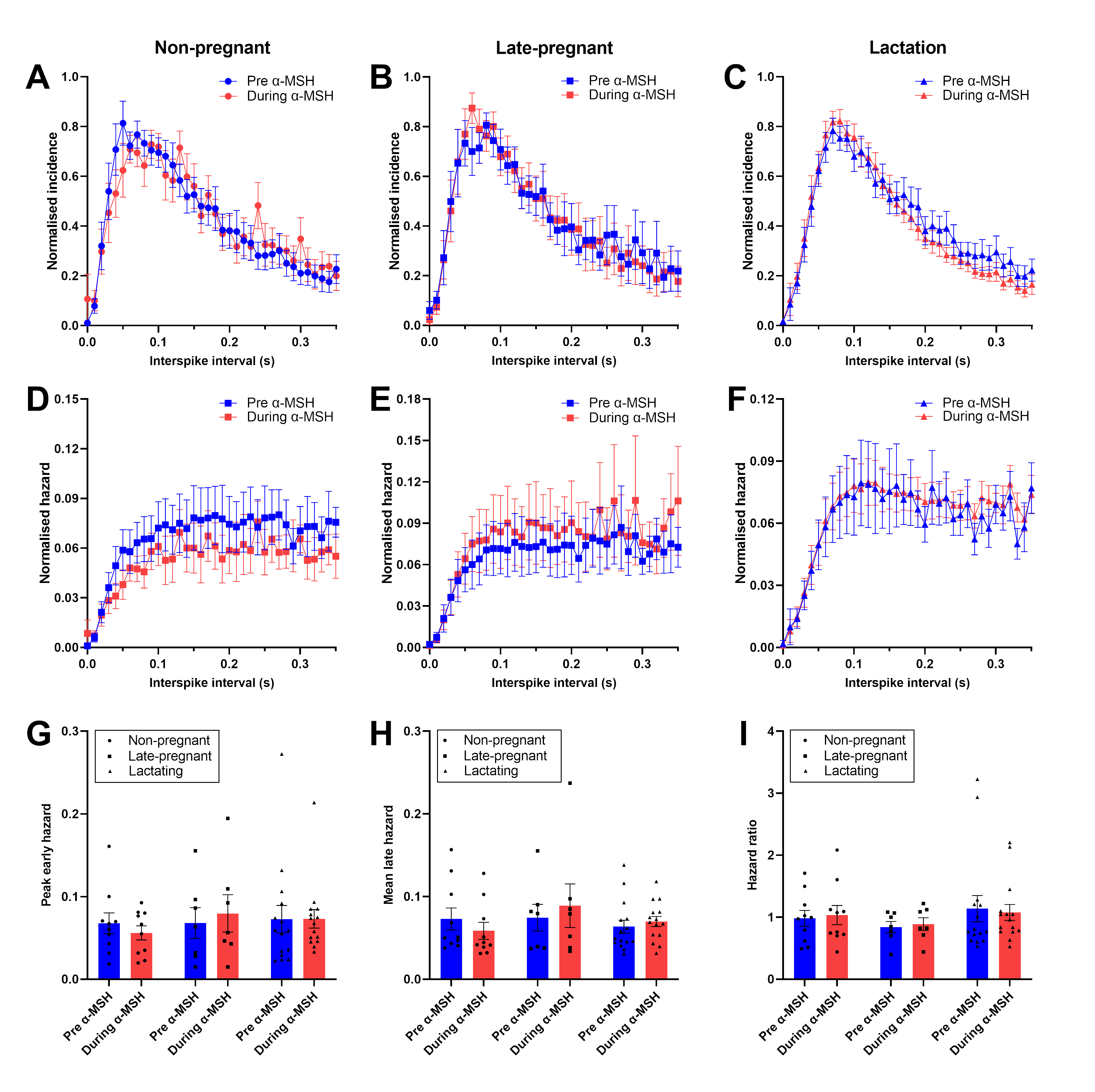

Supplement: Supplementary file 1 — Fig S1 [file PHY2-10-e15226-s001.tif]

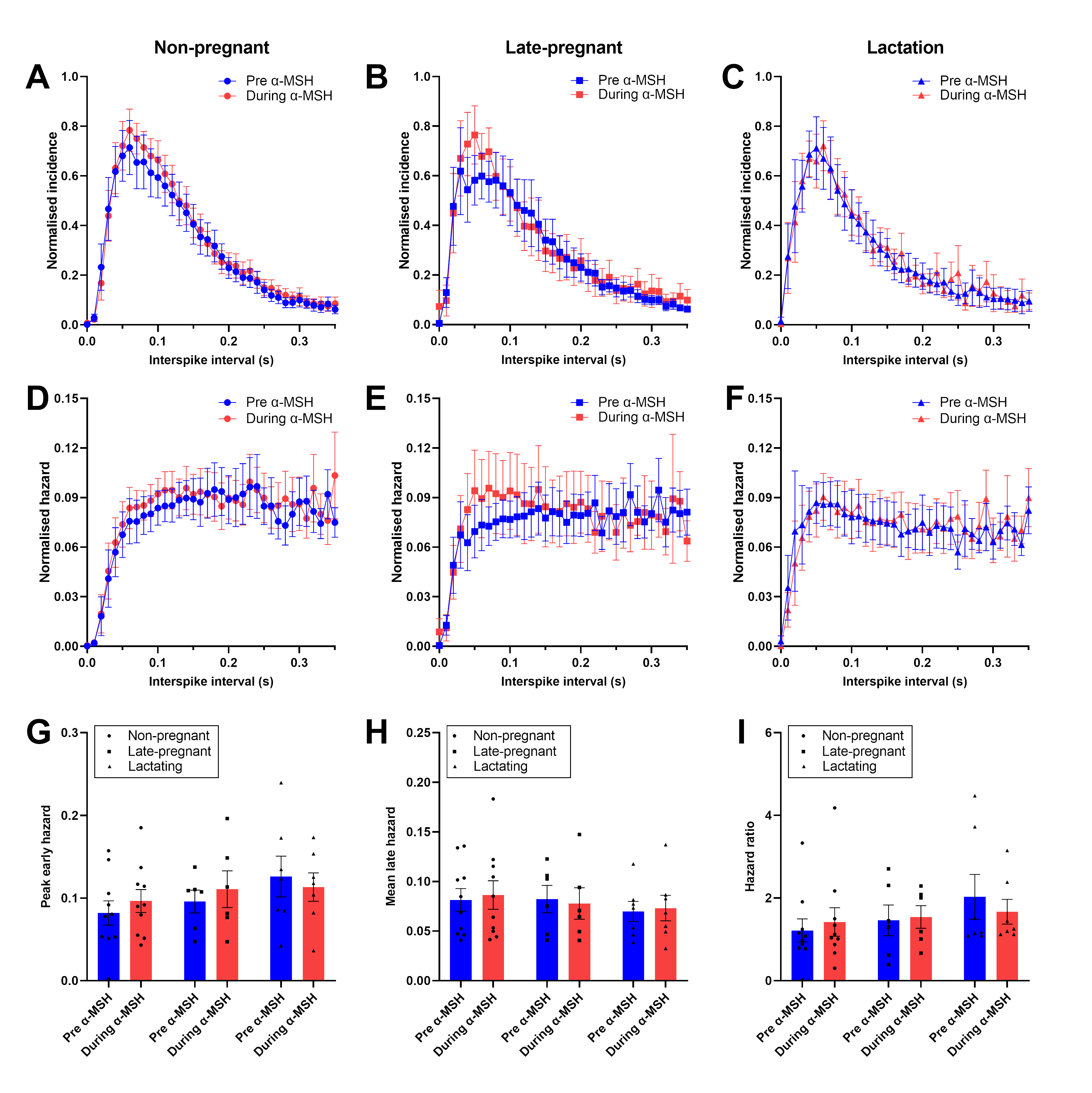

Supplement: Supplementary file 2 — Fig S2 [file PHY2-10-e15226-s002.tif]
